# AN AGE-FRIENDLY APPROACH TO THE DESIGN OF WEB-BASED USER INTERFACES

**DOI:** 10.1093/geroni/igae098.1541

**Published:** 2024-12-31

**Authors:** Justin Lam

**Affiliations:** Zucker School of Medicine at Hofstra/Northwell, Hempstead, New York, United States

## Abstract

Older adults are increasingly adopting digital technologies, with the majority of adults ages 65 and over being Internet users. These technologies can help support older adults’ well-being by improving access to and engagement with healthcare information. However, digital accessibility may continue to be an obstacle for adults who experience age-related functional changes, such as loss of vision and hearing or decline in cognitive and motor skills. While guidelines do exist for ensuring accessibility among people with disabilities, these guidelines do not always provide older adults with positive user experiences. A variety of frameworks have been proposed to support age-friendly user engagement with websites and mobile applications, with several centered on improving comprehension of text, location of information, and recognition of access links. In this presentation, we will explore the nuanced needs of users across different age groups, with special consideration given to the design of interfaces that can support both care partners and older adults. This presentation will cover a range of topics, such as optimizing user interaction patterns and enhancing readability, to provide practical insights into the concept of age-friendly design for user interfaces.

